# Thank you to our 2017 reviewers!

**Published:** 2018-05-31

Jacques Abourbih

Jonathan Ailon

Khaled Almansoori

Muneer Babar

Brenna Bath

Gisele Bourgeois-Law

Pamela Brett-MacLean

Allison Brown

Ford Bursey

Lita Cameron

Paula Cameron

Jessica Campoli

Dean Carson

Teresa Chan

Lawrence Cheung

Gerry Cooper

Janet Corral

John Dabous

Marlon Danilewitz

Andrea Davila Cervantes

Russell Dawe

Shafik Dharamsi

Tim Dube

Michael Epstein

Alison Eyre

Patrick Fleming

Sheri Fong

Alice Fornari

Wendy Fortna

Kristin Fraser

Isabelle Gauthier

Samantha Halman

Tayyab Hasan

Lara Hazelton

Nandita Hazra

Timothy Hierlihy

Carolyn Hoessler

John Hogenbirk

Joan Horton

Paul Humphries

Fred Janke

Aaron Jattan

Mary Johnson

David Kandiah

Rabia Khan

George Kim

Neils Koehncke

Lydia Kyei-Blankson

Andrew Latus

Karen Leslie

Fok-Han Leung

Ray Lewkonia

Lawrence Loh

Stuart Lubarsky

Marion Maar

Angela McGibbon

Meredith McKague

Scott McLeod

Gurpreet Narang

Alasdair Nazerali-Maitland

Stephanie Nishimura

Joyce Nyhof-Young

Jennifer O’Brien

Mini Pakkal

Robert Paul

Derek Puddester

Darryl Quinlan

Christen Rachul

Malathi Raghavan

Vivian Ramsden

Marghalara Rashid

Saleem Razack

David Rojas

Shelley Ross

Kathryn Roth

Katherine Rouleau

Anne Rowan-Legg

Todd Sakakibara

Kamran Sattar

Nicole Sbrocca

Michael Schwandt

Christine Seabrook

Stefanie Sebok-Syer

Noman Shahzad

Malika Sharma

Alexander Singer

Tom Smith-Windsor

Lyn Sonnenberg

Wendy Stewart

Ulrich Teucher

Maureen Topps

Eli Tshibwabwa

Anouk Utzschneider

Albina Veltman

Mohamud Verjee

Gary Viner

Hedy Wald

Andrew Warren

Marjorie Wenrich

Roselyn Wilson

Paul Winwood

Eric Wooltorton

Elizabth Wooster

Euson Yeung

Mary Zanetti

Mohammad Zubairi

